# Schmallenberg Virus among Female Lambs, Belgium, 2012

**DOI:** 10.3201/eid1907.121768

**Published:** 2013-07

**Authors:** François Claine, Damien Coupeau, Laetitia Wiggers, Benoît Muylkens, Nathalie Kirschvink

**Affiliations:** Namur Research Institute for Life Sciences, University of Namur, Namur, Belgium

**Keywords:** Schmallenberg virus, *Orthobunyavirus*, *Bunyaviridae*, *Culicoïdes*, midges, sheep, reemergence, arthrogryposis, viruses, vector-borne infections, lambs, Belgium

## Abstract

Reemergence of Schmallenberg virus (SBV) occurred among lambs (n = 50) in a sheep flock in Belgium between mid-July and mid-October 2012. Bimonthly assessment by quantitative reverse transcription PCR and seroneutralization demonstrated that 100% of lambs were infected. Viremia duration may be longer in naturally infected than in experimentally infected animals.

During late summer and fall 2011, a nonspecific febrile syndrome characterized by hyperthermia, decreased milk production, and diarrhea occurred among lactating cows in Germany. A new virus, named Schmallenberg virus (SBV), was identified as the cause (*1*). This arbovirus of the genus *Orthobunyavirus*, family *Bunyaviridae*, affects domestic and wild ruminants and has been documented in Western European countries since 2011 (*2*). The most notable consequences of this new pathogen are caused by its ability to cross the placental barrier. Depending on the gestational age of the offspring, abortion, stillbirth, or severe congenital malformations, including arthrogryposis and defects of central nervous system, might occur (*3**,**4*). Transplacental infection of offspring that occurred during 2011 led to economic losses in animal husbandry of sheep, goats, and cattle during birthing periods occurring during November 2011 through spring 2012 (*5**,**6*).

Several vectors of SBV have been identified. Biting midges, small flying insects of the species *Culicoides*, were vectors for serotype 8 of bluetongue virus that emerged during 2006 in Europe (*7*), and they seem to play a key role in spreading SBV (*8**,**9*). Similar to distribution of serotype 8 of bluetongue virus in 2007 (*10*), SBV circulation occurred during 2012 in regions where viral circulation was limited or not yet detected in 2011 (*11*). However, few investigations of acute viral circulation in regions where most of the ruminant livestock were infected during 2011 have been performed. The high in-flock seroprevalence ranging from 70–100% in regions documenting SBV outbreaks (i.e., North Rhine-Westphalia in Germany, the Eastern part of Belgium, and the southern part of the Netherlands) is believed to limit reemergence of SBV (*12*).

The objective of this study was to assess whether SBV reemergence occurred in a sheep flock that had experienced an SBV infection outbreak during autumn 2011 and reached a seroconversion rate of 99.5% (*13*). Female lambs born in late autumn 2011 or early winter 2012 were followed bimonthly to assess natural SBV primary infection by using quantitative reverse transcription PCR (RT-qPCR) and seroneutralization (SN).

## The study

Sheep of a flock that belonged to the University of Namur that included ≈400 ewes (Ile de France, Laitier Belge, French Texel, and crossbred) and ≈20 rams were investigated. During the lambing period of January 2012, 28 (17%) of 163 newborn lambs showed signs of congenital SBV infection that was confirmed by real-time RT-qPCR and SN assay (*13*). Two SBV strains were isolated in cell culture of brain tissue samples from congenitally infected lambs (*14*). Retrospective analysis of sentinels’ serum samples collected monthly in 2011 indicated that SBV infection of the flock occurred after September 15, 2011. SN assays were performed for all animals (n = 450) of the flock in January 2012 and revealed a seroprevalence of 99.5% (*13*).

Fifty female lambs born in autumn 2011 (n = 38) and January 2012 (n = 12) that were kept for breeding purposes were investigated by analyzing bimonthly blood samples collected during April–October 2012. The investigation protocol was approved by the Ethical Committee for Animal Welfare of the University of Namur (project 12/185). The animals were kept on pasture and underwent daily visual inspection. Blood was collected bimonthly and serum samples were analyzed by real-time RT-qPCR developed at the Friedrich Loeffler Institute (*1*) and by SN assay. RT-qPCR was performed on total RNA extracted from serum stored at –80°C. SN was performed after overnight incubation of a series of 2-fold dilutions for each serum sample with 100–200 tissue culture infectious dose_50_ of SBV (isolate SBV-BH80/11–4). Virus back titration was performed. The data were respectively expressed as cycle threshold (C_t_) and log_2_ 50% effective dose values. Results were considered positive at C_t_<40 and log_2_ 50% effective dose >3.5.

**Figure 1:**
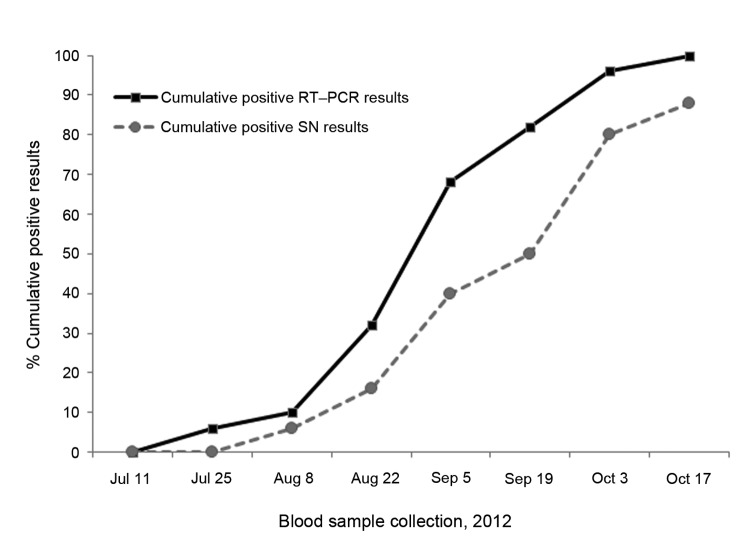
Time course of Schmallenberg virus spread among 50 infection-naive female lambs assessed bimonthly by real-time quantitative reverse transcription PCR (RT-qPCR) and seroneutralization (SN). Cumulative positive results (cycle threshold <40 and log_2_ 50% effective dose >3.5) obtained during July–October 2012 are expressed as percentages.

As shown in Figure 1, the SBV infection of the sentinels evidenced by RT-qPCR was first detected around mid-July and ended in mid-October 2012. Before this period, RT-qPCR results were negative and decrease of colostrum derived antibodies was observed (data not shown). Few animals were infected in July (3 animals were positive by using RT-qPCR on July 27) and at the beginning of August. Of the positive RT-qPCR results, 80% were found between mid-August and late September. By October 17, >1 positive RT-qPCR result had been detected for each animal (median C_t_ 37.03; minimum 29.4, maximum 39.5). SN assay revealed positive results for SBV antibody titers within 2–4 weeks after infection evidenced by RT-qPCR results. These findings are similar to observations made after experimental and natural SBV infection in cattle (*1**,**10*). During the period of monitoring, no clinical signs were detected.

**Figure 2:**
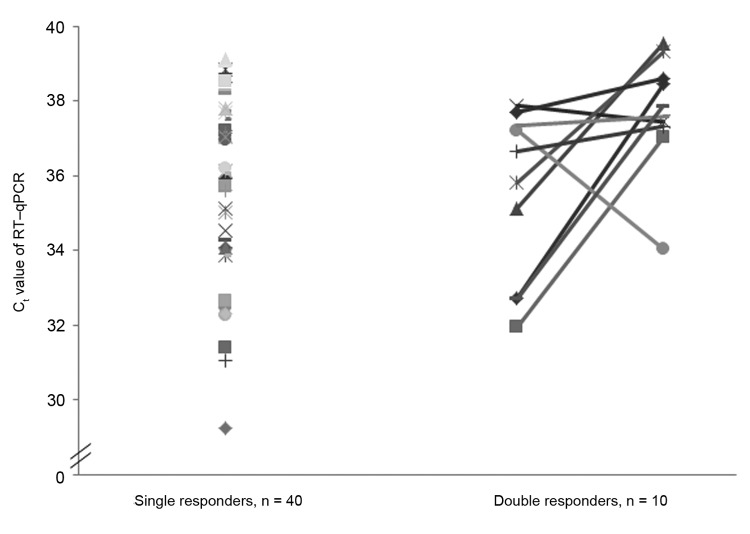
Individual cycle threshold (C_t_) values of real-time quantitative reverse transcription PCR (RT-qPCR) in 50 female lambs at time of natural infection by Schmallenberg virus. C_t_ values of lambs that tested positive once (single responders, n = 40) and those of lambs that tested positive twice during a 2-week interval (double responders, n = 10) are shown. Each symbol represents 1 lamb.

Against all expectations, all animals were positive for SBV at least once by using RT-qPCR. Ten lambs (20% of the investigated population) tested positive at 2-week intervals (Figure 2). This unexpected finding indicates that the duration of viremia in sheep (assessed as positive RT-qPCR result) may be longer after natural SBV infection in comparison to experimental SBV infection in cattle (*1*).

Although further investigations of a larger number of animals are needed, these preliminary results suggest that the time course of SBV infection depends on the age of the lambs (Table). The onset of SBV infection among lambs born in January 2012 occurred with a delay of 1 month. This finding is likely to be caused by the decrease of colostral protection.

**Table:** Time course of Schmallenberg virus spreading among female lambs born in late autumn and in January 2013 assessed by RT-q PCR and seroneutralization *

Date of blood sampling	2012								
	Jul 11	Jul 25	Aug 8	Aug 22	Sep 5		Sep 17	Oct 3	Oct 17
	No. (%) cumulative positive RT-qPCR results at each sampling date								
Lambs born in autumn 2012†	0	3 (8)	5 (13)	11 (29)		25 (66)	30 (79)	36 (95)	38 (100)
Lambs born in January 2013‡	0	0	0	5 (42)		9 (75)	11 (92)	12 (100)	12 (100)
	No. (%) cumulated positive seroneutralization results at each sampling date								
Lambs born in autumn 2012†	0	0	3 (8)	8 (21)		18 (47)	22 (58)	33 (87)	35 (92)
Lambs born in January 2013‡	0	0	0	0		2 (17)	2 (17)	6 (50)	8(67)

*Cumulated positive results obtained between July and October 2012 are expressed as absolute numbers and percentages.RT-qPCR results were considered positive if cycle threshold value was <40. Seroneutralization results were considered positive if log_2_ 50% effective dose values were > 3.5. RT-qPCR,real-time reverse transcription quantitative PCR. †n=38. ‡n=12.

The data collected during this study demonstrate that, even in regions with a high SBV seroprevalence, viral circulation and primary infection of naive animals occur. In addition to the question of duration of colostral protection, this observation raises questions about virus hosts. It might be hypothesized that other species are reservoirs. In autumn 2011, a seroprevalence of ≈43% was found in deer in the province of Namur (*15*), suggesting that deer might have been a reservoir during 2011–2012. Serum samples of 3 roe deer hunted in autumn 2012 in the forest near the sheep flock observed in this study tested positive by SN assay (N. Kirschvink, pers. comm.), indicating a higher percentage of seroconversion among samples from deer in 2012. It might further be speculated that notable vector activity favored viral circulation. During the summer 2007 serotype 8 of bluetongue virus outbreak, vector activity was assessed among the study flock of sheep and revealed substantial numbers of newly emerged female *Culicoides* spp. midges (*7*). This peak activity of vectors paralleled the episode of clinical serotype 8 of bluetongue virus manifestation. Hypothesizing that the local *Culicoides* spp. midge population did not substantially change over years, the intense infection rate during August–September 2012 could be related to increased seasonal vector activity.

Although reemergence of SBV among cattle is of concern, reemergence of SBV among sheep and goats is of particular importance, because high prolificacy and intensive reproduction of sheep and goats continuously lead to a noteworthy percentage of susceptible animals among the population. Moreover, the early onset of puberty in these species increases the probability of SBV infection at an early stage of gestation, leading to potential virus overwintering by transplacental infection of offspring, which could potentially lead to economic loss related to death or culling of offspring. Detailed knowledge about duration of viremia after natural SBV infection and duration of colostral protection are necessary for elaboration of efficient breeding and vaccination strategies.
